# Open and Calm – A randomized controlled trial evaluating a public stress reduction program in Denmark

**DOI:** 10.1186/s12889-015-2588-2

**Published:** 2015-12-16

**Authors:** Christian G. Jensen, Jon Lansner, Anders Petersen, Signe A. Vangkilde, Signe P. Ringkøbing, Vibe G. Frokjaer, Dea Adamsen, Gitte M. Knudsen, John W. Denninger, Steen G. Hasselbalch

**Affiliations:** Neurobiology Research Unit (NRU) and Center for Integrated Molecular Brain Imaging (Cimbi), The Neuroscience Centre, Rigshospitalet and University of Copenhagen, Juliane Maries Vej 28, 3rd floor, 2100 Copenhagen OE, Denmark; Center for Visual Cognition, Department of Psychology, University of Copenhagen, Øster Farimagsgade 2A, 1353 Copenhagen K, Denmark; Benson-Henry Institute of Mind-Body Medicine, Massachusetts General Hospital, Boston, USA; Danish Dementia Center, Copenhagen University Hospital, Copenhagen, Denmark

**Keywords:** Stress reduction, Mental health promotion, Meditation, Cortisol, Attention

## Abstract

**Background:**

Prolonged psychological stress is a risk factor for illness and constitutes an increasing public health challenge creating a need to develop public interventions specifically targeting stress and promoting mental health. The present randomized controlled trial evaluated health effects of a novel program: Relaxation-Response-based Mental Health Promotion (RR-MHP).

**Methods:**

The multimodal, meditation-based course was publicly entitled “Open and Calm” (OC) because it consistently trained relaxed and receptive (“Open”) attention, and consciously non-intervening (“Calm”) witnessing, in two standardized formats (individual or group) over nine weeks. Seventy-two participants who complained to their general practitioner about reduced daily functioning due to prolonged stress or who responded to an online health survey on stress were randomly assigned to OC formats or treatment as usual, involving e.g., unstandardized consultations with their general practitioner. Outcomes included perceived stress, depressive symptoms, quality of life, sleep disturbances, mental health, salivary cortisol, and visual perception. Control variables comprised a genetic stress-resiliency factor (serotonergic transporter genotype; 5-HTTLPR), demographics, personality, self-reported inattentiveness, and course format.

**Results:**

Intent-to-treat analyses showed significantly larger improvements in OC than in controls on all outcomes. Treatment effects on self-reported outcomes were sustained after 3 months and were not related to age, gender, education, or course format. The dropout rate was only 6 %.

**Conclusions:**

The standardized OC program reduced stress and improved mental health for a period of 3 months. Further testing of the OC program for public mental health promotion and reduction of stress-related illnesses is therefore warranted. A larger implementation is in progress.

Trial registration: ClinicalTrials.gov.: NCT02140307. Registered May 14 2014.

**Electronic supplementary material:**

The online version of this article (doi:10.1186/s12889-015-2588-2) contains supplementary material, which is available to authorized users.

## Background

Public health sectors in modernized countries are burdened by growing reports of prolonged, psychosocial stress. Otherwise healthy individuals experience that the demands of the environment (most often their occupation) exceed their available resources to a degree that disrupts their daily functioning by way of e.g., concentration problems, irritability, anxiousness, depressive symptoms, fatigue, or bodily pain. About a fourth of North Americans regularly experience high levels of stress [2]. In Denmark, such estimates increased from 6 % in 1987, to 9 % in 2005, and 15 % in 2012 [22, 38]. Prolonged stress is associated with impairments of the cardiovascular, immune, metabolic and nervous systems [56]. For example, long-term psychosocial stress is related to significant increases in neurological inflammatory processes [51], and with increased risk for depression [37]. Recent research also connects stress to sleep disturbances [66].

For these reasons, health agencies have underlined a public need for evidence-based programs specifically targeting psychosocial stress and promoting stress resiliency [84]. This was also governmentally reinforced in Denmark [7]. Unfortunately, only about 5 % of Danish health research concerns public health [36].

Therefore, we developed a program designed for stressed, but otherwise healthy adults to reduce stress and promote mental health and resiliency. Reviews have documented that meditation-based multimodal programs reliably reduce stress in healthy samples [21, 33, 68]. However, meditative programs are generally modeled on complex philosophical-religious systems and not academic theories [68]. As an exception, the so-called Relaxation Response (RR) research tradition lead by Herbert Benson and colleagues has through four decades provided empirical evidence supporting that a few core methodological commonalities are evident across many contemplative traditions’, and that regular practicing of these techniques is sufficient for eliciting physiological stress reduction and for improving overall health [65]. In targeting a broad demographic group, and since we aimed to develop a theoretically driven and methodologically consistent and well-defined program, we selected RR-based meditation. For the same reasons, we structured the course content according to the well-established body-psycho-social understanding of stress (e.g., [56]). Finally, a novel, cognitive framework model termed “Open and Calm” (OC) was used every week to integrate the meditative, bodily, cognitive, and social practices.

Our primary hypotheses were that OC would reduce self-reported perceived stress as well as physiological stress as measured by cortisol secretion upon awakening [30]. Based on longitudinal studies suggesting that a blunted HPA-axis response to awakening develops with prolonged distress over time [6] and on several studies associating burnout with blunted HPA-axis reactivity [43, 54, 59, 67], we held the secondary hypothesis that intervention participants with blunted baseline cortisol secretion curves upon awakening would exhibit a reestablishment of HPA-axis reactivity. Oppositely, stressed intervention participants with non-blunted cortisol reactivity were predicted to decrease their cortisol awakening response relative to controls. However, HPA-axis dysregulation in relation to prolonged stress and burnout is complex and not fully understood [25]. In further secondary hypotheses, we stated that OC would improve self-reported mental health, quality of life, symptoms of depression, and sleep disturbances, as well as visual attention, as argued by theories of mechanisms of change in meditation [4, 14].

It was also theoretically important to investigate several potential treatment effect moderators: First, carriers of S and LG alleles in the serotonin transporter-linked polymorphic region (5-HTTLPR) of the SCL6A4 gene [16] show increased risk for depression after severe stress in most population studies [46], as well as increased cortisol response to stressors [20, 60]. Second, the personality trait “harm avoidance”, reflecting a proclivity to repress stressful stimuli, may decrease stress resiliency, while increased “self-directedness”, reflecting overall top-down self-regulation abilities, may promote stress resiliency [72]. Third, variables such as age, gender, and education are recommendable covariates in public health promotion to evaluate the demographic applicability of the intervention [45].

## Method

### Participants

**Fig. 1:**
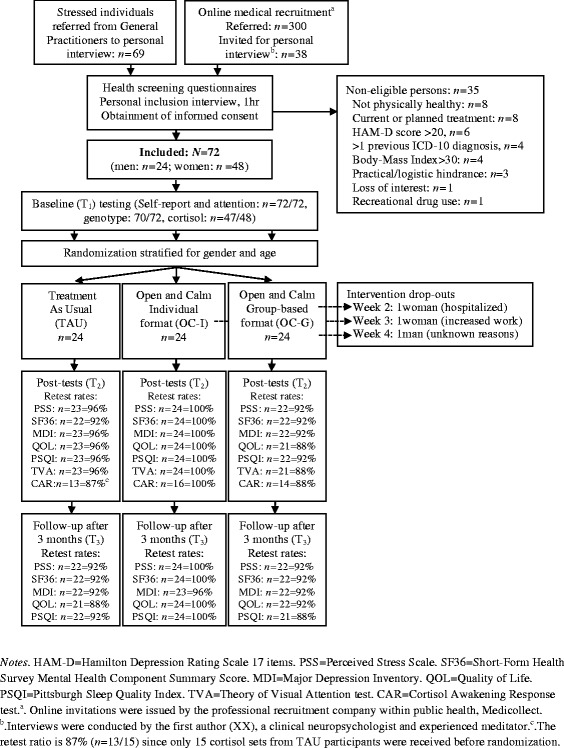
Participant flow in the Open and Calm Randomized Controlled Trial

Participants were stressed, but otherwise healthy, Danish adults (65 % women) aged 18–59 years (Mean = 42 years, Standard Deviation = 9 years, interquartile range: 36–48). Participants seldom reported to have no professional education, and relatively often to have longer professional educations, compared with the Copenhagen adult population at the time [70] (% of sample/% of population: no professional education: 8 %/33 %; apprenticeship: 24 %/22 %; 1–3 years: 14 %/5 %; bachelor degree or 3–4 years: 24 %/22 %;>4 years: 31 %/19 %). All were Caucasian. The majority (92 %) never meditated regularly (>2 times/week for > 1 month) before. Additional file 1: Table S1 provides more detailed sample characteristics. The inclusion criteria were the age 18–59 years, fluency in Danish, and subjective report of reduced daily functioning due to stress for more than one month. This was evaluated in a 1-hr personal inclusion interview (Fig. 1 shows the participant flow). The main exclusion criteria were current treatment for any illness; >1 diagnosed or treated ICD-10 mood disorder (F30-39) or any other ICD-10 disorder for adults within three years; Hamilton Depression Rating Scale score >20 at the inclusion interview; recreational drug use >24 times per year or > 50 times in the lifetime; Body-Mass-Index (BMI) >30 (due to exploratory psycho-physiological measurements), and medication use affecting the brain or cortisol, such as selective serotonin reuptake inhibitors or corticosteoroid medications.

### Procedures

The present Clinical Registered Trial (Clinicaltrials.gov ID: NCT02140307), approved by the Danish Ethics Committee (H-3-2012-092), recruited volunteers through 20 General Practitioners (GP) and an online medical recruitment company. Figure 1 shows participant flow and retest rates. Participants provided informed consent. Stratifying for age and gender, the last author SGH, who had no participant contact, block-randomized three consecutively enrolled cohorts of *n* = 24 to intervention in individual format, group-format, or treatment as usual (TAU), involving e.g., extra GP visits, or stress leave. Groups were randomized with a ratio of 1:1:1 using www.random.org. An *a priori* power calculation in G-power [29] revealed a required *N* = 54 (power = .95, three groups, three measurements [pre, post, follow-up], expected effect *f =* 0.25, sphericity correction = 1). This power analysis did not specify the number of expected covariates, since the theoretical knowledge on effect moderators in meditation-based stress reduction programs is very limited [35], especially for healthy samples [68]. A medium effect size after meditation-based stress reduction programs is commonly found on self-report outcomes [68]. Effect sizes for behavioral and physiological outcomes vary more in studies of meditation-based stress reduction [55], but we have previously demonstrated medium-large effects of mindfulness-based stress reduction (MBSR; [44]) on the presently investigated physiological and perceptual tests and outcomes [42]. Expecting 15–30 % dropout [64], *N* = 72 were therefore recruited.

Danish, validated self-report instruments were completed online at home. Double-blinded baseline data (T_1_, Jan.–Mar., 2013) were obtained before randomization (Fig. 1). To increase validity, scales were completed both at 4 weeks and 2 weeks pre-intervention and 2 weeks and 4 weeks post-intervention (Apr.–Jun, 2013). T_1_ and T_2_ scores reflect the average of each pair of completions, as recommended [80]. Cortisol (for financial reasons only collected for the first *n* = 48) and attention were tested within 2 weeks before and after the intervention period by researchers blinded to participant status at T_2_. Follow-up 3 months after the intervention (T_3_, Oct.–Dec., 2013) included self-report. Participants were not contacted during the follow-up period itself.

### The intervention

The “Open and Calm” (OC) program was based on the Relaxation Response (RR) tradition [65], which has for decades empirically supported that many meditative techniques elicit the same physiological, parasympathetic RR, involving e.g., lowered heart rate, blood pressure, and respiration rate. RR theory proposes that the core methods across meditative techniques necessary for eliciting the RR are: (a) the continuous returning of attention to a meaningful focus, i.e., focused attention training, and (b) the non-reactive or contemplative witnessing of ongoing experience. The OC meditation focused on these aspects and termed them “Open” (relaxed and receptive attentiveness) and “Calm” (non-intervening witnessing).

The course structure was modeled on the well-established overall understanding that bodily (biological), psychological, and social factors interact in stress, stress resiliency, and health, focusing each week on working with either the body, the mind, or social relationships, in a cyclic fashion. Meditation was trained every week; bodily, cognitive, and social practices followed the weekly themes. Importantly, all practices focused on training the OC states (e.g., Open attention toward the breath, an emotion, or another person) and were theoretically integrated by a core OC cognitive framework model.

The OC program is standardized [40] and was offered in two formats: The group format (OC-G) involved weekly 2.5-hr group sessions (n = 8 per group) and two optional 1.5-hr personal sessions. The individual format (OC-I) involved personal, weekly 1.5-hr sessions. Formats used identical materials, e.g., a 120-page course book [39], online materials, 1–2 daily meditations of 10–20 min following audio files, and frequent “mini-meditations” of 1–2 min.

## Measures

### Control variables

#### Demographics and life style

Demographic factors investigated as covariates included age, gender, education, and occupational status (employed/unemployed). Life style variables included previous meditation experience, alcohol consumption, tobacco use, and BMI.

##### Genotype

Saliva was collected in DNA Genotek tubes (Ottawa, Canada) and frozen at −80 degrees Celsius until analyzed. Polymerase chain reaction (PCR) was used to amplify the 5-HTTLPR and two oligonucleotide primers [83] to generate allele-specific fragments: short (S) allele 469 bp and long (L) allele 512 bp. PCR was performed in a GeneAmp PCR System 9700 (Applied Biosystems MspI). The genotype covariate quantified the efficiency of 5-HT reuptake: 0 = SS/SLG*, n* = 14; 1 = SLA/LGLA, *n* = 41; 2 = LALA, *n* = 15; missing: *n* = 2.

##### Personality

From Temperament and Character Inventory (TCI) [23] the personality trait *Harm Avoidance* (TCI-HA) evaluated the proclivity to avoid novelty, non-reward and punishment. The trait *Self-Directedness* (TCI-SD) measured executive, self-regulation and adaption. TCI-HA and TCI-SD were recommended as screening tools in public health studies of stress [72]. Both factors were internally consistent, Cronbach’s *α*s ≥ .84.

##### Stressful life events

Stressful Life Events (SLE; [47]) was used to investigate SLE within the past year and the lifetime (e.g., assault, job loss, serious illness, loss of a confidant).

##### Attentional instability

Mindful Attention Awareness Scale (MAAS; [11]) evaluated attentional instability at baseline via 15 items and was internally consistent, *α* = .88. The Danish translation of the MAAS has been psychometrically validated [41].

##### Test motivation

At each cognitive test, participants rated how motivated they were to comply with the task on a 7-point Likert-scale from 0 (not at all motivated) to 6 (very motivated).

##### Course attendance

Course length or the number of treatment days [17, 68, 81], as well as the degree of compliance with meditation pratices [74, 79] have not shown consistent relationships with effects of mindfulness-based programs for stress reduction or health promotion. Similarly, a comprehensive review of an RR-based stress reduction program found that treatment effects on stress-related outcomes (anxiety and hostility) were not related to pre-post changes in weekly meditation practices [18]. To minimize participant burden and lower dropout, compliance was presently only quantified as the number of attended OC sessions.

### Outcome variables

#### Perceived stress

Cohen’s Perceived Stress Scale (PSS; [24]) comprise 10 items of stress-related experiences rated from 0 (never) to 4 (very often) for their frequency during the past two weeks, providing an overall score. The PSS was always internally consistent, *α*s ≥ .82.

##### Mental health

Short-Form Health Survey-36 (SF-36; [82]) measures eight health dimensions: 1) physical function, 2) physical role limitations, 3) bodily pain, 4) general health, 5) emotional function, 6) vitality, 7) emotional role limitations, and 8) mental health. Each dimension is scored from 0 (poor) to 100 (best possible). The Mental Component Summary score (SF-36-MCS) was based on weighting of all dimensions [5]. At all times, *α* was ≥ .71.

##### Depressive symptoms

Major Depression Inventory (MDI; [3]) involve ratings of the frequency of the ten ICD-10 depressive symptoms during the past two weeks (0 = not at all, 5 = all of the time). The total MDI was investigated and was always internally consistent, *α*s > .83.

##### Quality of life

The 5-items Quality of Life (QOL) developed by WHO assesses quality of life through positive affect and vitality. The Danish QOL has high validity and QOL scores <50 is a risk marker for depression [31]. QOL was internally consistent, all present *α*s > .81.

##### Sleep disturbances

Pittsburgh Sleep Quality Index (PSQI; [15] indexes sleep disturbances during the past month via 19 items. On the examined PSQI Global, scores >5 indicate increased risk for depression. Consistency was mostly satisfactory, *α*s: T_1_ *=* .61; T_2_ = .77*;* T_3_ = .69.

##### Physiological stress

The cortisol awakening response (CAR) reflects hypothalamic–pituitary–adrenal (HPA) axis activity [30]. After written and verbal instructions and training, participants performed home-samplings of saliva in Salivette tubes (Sarstedt, Neubringen, Germany). Sample 1 was taken immediately upon awakening, and samples 2–5 every 15 min for the subsequent hour. Participants registered the time of awakening and of each sampling. Within 48 hrs samples were centrifuged and stored at –80 degrees Celsius. The entire batch was analyzed in one step using electrochemiluminescent immunoassay (Cobas equipment, Roche, Germany). Our outcomes were the *Area Under the Curve with respect to ground* (AUC_G_), representing the total magnitude of cortisol secretion; and the *Area Under the Curve with respect to increase from awakening levels* (AUC_I_), reflecting the HPA axis’ cortisol response to awakening [30]. Participants with symptoms of burnout at T_1_ (blunted CAR [AUC_I_] curves) were analyzed separately. Blunted T_1_ CAR curves were identified by inspection of individual curves by two researchers (anonymized, anonymized) blinded to participant group status.

##### Visual attention

The computational Theory of Visual Attention (TVA; [12]) framework quantifies functions of visual attention using accuracy-based testing. The TVA-based test used here (ad modum [77]) comprised two practice blocks and three test blocks of 30 trials presenting six red letters on a computer screen. The letter display durations were varied systematically (20–200 ms), and terminated by pattern masks (500 ms) before participants made an unspeeded report of letters they were “fairly certain” of having seen. In cognitive test rooms, participants were instructed to refrain from pure guessing and to aim for an accuracy of 80–90 %. They were informed about their accuracy after each block. Three parameters of attention were extracted by mathematical modeling [28]: The storage capacity of visual short-term memory (*K*; 5 degrees of freedom [df]), the speed of visual processing (*C*; 1 df), and the threshold for conscious visual perception (*t*_0_; 1 df). Since meditation may specifically improve visual perceptual thresholds [42, 52], *t*_0_ was our visual attention outcome, while *K* and *C* analyses were exploratory.

### Statistical analyses

Intent-To-Treat (ITT) models were applied, replacing missing T_2_ or T_3_ scores with T_1_ or T_2_ scores, respectively. Group differences in outcome changes were investigated in two-way repeated measures ANCOVAs using Time (T_1_/T_2_/T_3_) and Group (e.g., OC/ TAU controls) as independent variables. These main analyses adjusted for covariates (see ‘Control Variables’) that correlated with (*p* < .05) outcome change scores (T_1_–T_3_) within the compared groups. Adjustment was carried out by including Time × Group × Covariate-interactions in the models. Continuous covariates were centered before entering the models [1, 73]. Significant Time × Group × Covariate-interactions were interpreted as indications of a potential effect-moderating role for the covariate [50, 76]. These interpretations were based on post hoc visual plots and correlation tests as described in the Results section.

Candidate covariates were selected based on theories and studies of mechanisms of change in meditation-based stress reduction, while the actual inclusion of covariates in the final analyses was data-driven. This strategy was applied because OC is a new program and because theories on moderators and mediators of change in meditation-based programs are preliminary and sparse [35, 48]. This is especially the case for studies healthy samples, which have “largely been conducted in a rather atheoretical manner” ([68], *p*. 1161). To promote progress in this area, we discuss potential effect moderators, but the present RCT focused on investigating the effectiveness of OC compared to TAU.

We explored different analytic approaches by also including covariates that were associated with (*p <* .05) outcome scores at baseline and by examining T_1_-T_2_-change scores’ associations with candidate covariates. We also tried excluding all scores >3.0 *SD* from group means (<2 % in all analyses). These analytic variations did not change any Time ^⊥^ Group results significantly. In the final analyses, one score was excluded, being a T_2_*t*_0_-value (0.7 %; replaced with the T_1_*t*_0_-value) of inadequate data quality. All *p*-values were Bonferroni-Holm-corrected for the number of group comparisons within each outcome type (self-report/cortisol/attention). The analysis of Time ^⊥^ Group interactions on depressive symptoms violated the assumption of sphericity (Mauchly’s *W* = 0.87, *p* < .05) and was thus Huyn-Feldt-corrected.

OC format was not expected to affect intervention effects [9, 53, 81], but this was investigated in an initial OC-I *vs.* OC-G comparison. If formats did not differ (*p <* .05), the collapsed OC was then compared to TAU controls. If formats did differ, each format was to be compared to controls in turn. A multivariate analysis of covariance (MANCOVA) examined whether gender, age (median split), or education (3 *df*) affected long-term (T_1_–T_3_) change scores across all self-report scales in OC. The MANCOVA was designed to test the socioeconomic applicability of OC and therefore did not include other candidate effect moderators, such as genotype or personality. To strengthen our interpretation, we followed up the MANCOVA with a series of zero-order post hoc correlations testing associations between each demographic factor (age, gender, and education) and short-term (T_1_–T_2_) as well as long-term (T_1_–T_3_) outcome changes, respectively.

Effect sizes were expressed with Cohen’s *d* (group differences and pre-post within group effects corrected for dependence among means ad modum [58]; formula 8), Pearsons *r* or Spearman’s *rho* (ρ) (variable associations), or partial eta-squared, *η*_*p*_^*2*^, (Time × Group effects and Time × Group × covariate interactions). MDI and PSQI data were skewed and log^10^-transformed, yielding normal distributions. Questionnaires’ internal consistency was assessed with Cronbach’s alpha, *α*. We pre-defined AUC_G_ and PSS as primary outcomes in the trial protocol. Similarly, we specified AUC_I_, SF-36-MCS, MDI, QOL, PSQI, and *t*_0_ as secondary outcomes. Analyses were carried out in SPSS (IBM, vs. 20.0) and Microsoft Excel 2011.

## Results

### Course attendance

OC had a 94 % (*n* = 45/48) completion rate. The three dropouts did not differ from other participants on any baseline characteristics. In total, group participants attended more sessions (mean[M] = 8.3, *SD* = 2.7) than individual format participants (M = 6.7, *SD* = 2.0) (*p* = .020), but required less (M = 3.9 hrs, *SD* = 1.7) professional contact hours per participant (M = 10.0 hrs, *SD* = 3.0), a ratio of 2.56. Session attendance rates were unrelated to outcome changes unless otherwise is stated.

### Self-report

**Fig. 2:**
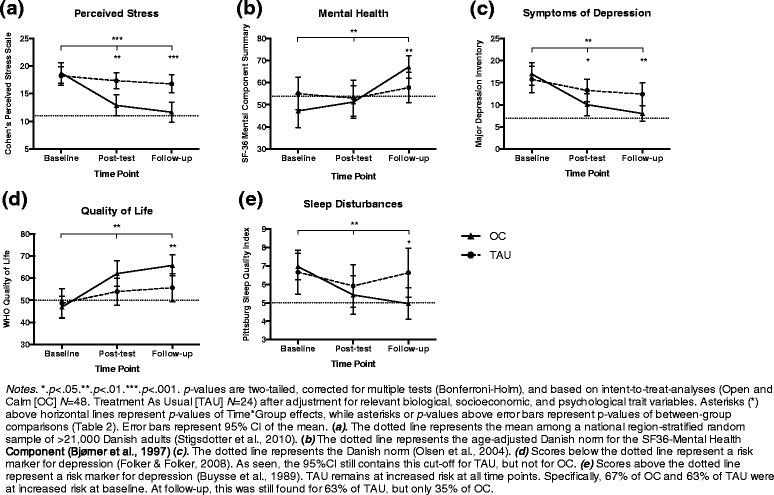
Group comparisons on self-report outcomes

Intervention format did not affect changes in the primary self-report outcome of perceived stress, PSS, *p* = .13), or any secondary self-report changes, *p*s > .1 (uncorrected *p*s ≥ .06; Additional file 2: Panel S1). The total intervention group improved significantly more than TAU controls on PSS, *p* < .0001. Similarly, OC improved significantly more than TAU on all secondary self-report scales, *p*s < .005 (Table 1 and Fig. 2). Effects were sustained or significantly improved on all scales during follow-up and OC differed significantly from controls on all scales at T_3_, *p*s < .02. OC increased above the quality of life risk marker for depression; controls did not (Fig. 2, d). OC decreased slightly below the sleep disturbances risk marker for depression; controls did not (Fig. 2 , e). The MANCOVA showed no effect of age, gender, or education across self-report effects for OC, *p* > .2. In line with this, age, gender, and education were unrelated with both short-term (T_1_–T_2_) and long-term (T_1_–T_3_) changes, respectively, on all self-report outcomes, *r*s ≤ .21, *p*s > .17. (See further details on potential effect moderators in Additional file 3: Supplementary findings—potential effect moderators).

**Table 1:** Treatment effects on self-report outcomes

Outcome	Open and Calm (OC)		Treatment as Usual (TAU)		OC vs. TAU	OC vs. TAU change^a^				
	M (SD)	d (within)	M (SD)	d (within)	d (between)	p	F	η_p_^2^ (between)	p	Covariates
Perceived Stress (PSS)										
Pre-treatment (T_1_)	18.75 (6.48)		18.22 (4.01)		0.09	.718	12.96	.16****	< .0001	
Post-treatment^b^ (T_2_)	12.88 (7.31)	0.92***	17.33 (3.51)	0.22	0.71**	.012				Employment TCI-HA
Follow-up^c^ (T_3_)	11.64 (6.26)	0.24	16.77 (3.83)	0.25	0.93***	.001				
Pre-treatment—Follow-up		1.30***		0.39*						
Mental Health (SF-36-MCS)										
Pre-treatment (T_1_)	47.24 (26.05)		55.06 (17.26)		0.20	.299	4.97	.13**	= .0018	
Post-treatment^b^ (T_2_)	51.22 (25.17)	0.21	52.95 (19.26)	−0.18	0.08	.971				Age
Follow-up^c^ (T_3_)	67.09 (17.57)	0.89***	57.73 (16.38)	0.30	0.55*	.012				
Pre-treatment—Follow-up		0.99***		0.15						
Depression (MDI)										
Pre-treatment (T_1_)	16.98 (8.67)		15.75 (7.10)		0.15	.551	8.01	.11**	= .002	
Post-treatment^b^ (T_2_)	10.04 (8.65)	0.91***	13.27 (5.97)	0.36	−0.42*	.044				MAAS
Follow-up^c^ (T_3_)	8.04 (6.01)	0.51	12.42 (6.02)	0.22	−0.74**	.006				
Pre-treatment—Follow-up		1.44***		0.60*						
Quality of Life (WHO-5)										
Pre-treatment (T_1_)	46.88 (17.32)		48.67 (15.72)		−0.11	.671	5.71	.08**	.004	
Post-treatment^b^ (T_2_)	62.04 (19.84)	1.01***	53.92 (14.54)	0.31	0.45	.080				BMI
Follow-up^c^ (T_3_)	65.75 (16.44)	0.23	55.67 (15.19)	0.10	0.64**	.014				TCI-HA
Pre-treatment—Follow-up		1.06***		0.39						
Sleep Quality (PSQI)										
Pre-treatment (T_1_)	6.97 (2.49)		6.67 (2.81)		0.12	.531	11.27	.14***	= .0001	
Post-treatment^b^ (T_2_)	5.43 (3.63)	0.47***	5.92 (2.73)	0.25	−0.15	.254				Smoking
Follow-up^c^ (T_3_)	4.96 (2.93)	0.22	6.63 (3.16)	−0.22	-.56*	.017				
Pre-treatment—Follow-up		0.73***		0.01						

*Notes*. *.*p*<.05.**.*p*<.01.***.*p*<.001. All *p*-values are two-tailed , Bonferroni-Holm corrected, and based on intent-to-treat-analyses (OC: *n*=48; TAU: *n*=24). The OC *vs*. TAU change-models adjusted for relevant biopsychosocial covariates (listed in the column “Covariates”) as well as for Time✕Group✕Covariate-interactions. ^a^. Effect sizes indicate pre-treatment—post-treatment—follow-up Time✕Group effects. ^b^. Within-group effect sizes indicate pre-treatment—post-treatment effects. ^c^. Within-group effect sizes indicate post-treatment—follow-up effects. MAAS = Mindful Attention Awareness Scale. MDI=Major Depression Inventory. PSQI=Pittsburgh Sleep Quality Index. PSS=Cohen’s Perceived Stress Scale. SF-36-MCS=Short Form Health Survey-36-Mental Component Summary. TCI-HA = Temperament and Character Inventory - Harm-avoidance. Smoking = Smokers was defined as participants who smoked daily, while other participants were defined as non-smokers

**Fig. 3:**
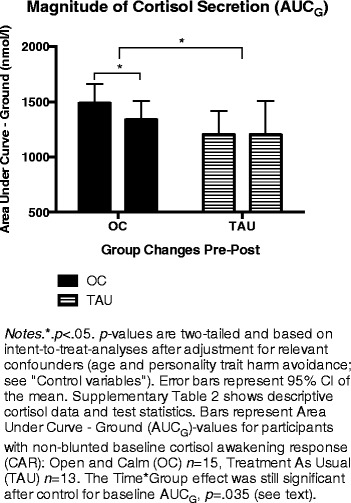
Changes in cortisol secretion

### Physiological stress

Cortisol outcomes were normally distributed in all groups at all time points, *p*s > .15 (Shapiro-Wilk). OC-I and OC-G did not differ at any time point or from pre-post treatment, *p*s > .09 (uncorrected). We then compared all OC participants to all TAU controls. In these primary analyses, the two groups did not differ on any cortisol outcomes at baseline, post-treatment, or in changes from pre-post (Additional file 4: Table S2). However, our secondary hypothesis stated that baseline CAR profile (blunted/non-blunted T_1_ CAR) would affect the directionality of CAR changes for OC participants. In support of this, OC participants with a non-blunted T_1_ CAR decreased significantly on AUC_G_, *p* = .018, *d* = −0.59 (Additional file 5: Panel S2; Additional file 4: Table S2). This decrease in OC was significantly larger than in non-blunted controls, *p* = .030 (corrected)*, η*_*p*_^*2*^ = .24 (Fig. 3; Additional file 4: Table S2). Since OC showed non-significantly higher baseline cortisol (AUC_G_) levels, we conducted a *post hoc* ANCOVA also controlling for baseline AUC_G_, and still controlling for TCI-HA, age, and the three interaction terms. This test reaffirmed that the treatment group decreased significantly more than controls (Time × Group interaction) *F*(1,20) = 5.09, *p* = .035, *η*_*p*_^*2*^ = .20. Group changes for AUC_I_ did not differ, but only OC decreased significantly, *p* = .018, *d* = −0.76 (Additional file 5: Panel S2, a). Blunted baseline CAR was identified for *n* = 18 in OC, and *n* = 2 in TAU. Group comparisons were therefore not meaningful for these AUC_I_ analyses. As we hypothesized, CAR-blunted OC participants showed a significantly increased AUC_I_, *p* = .015, *d* = 0.88 (Additional file 5: Panel S2, b). This effect suggested a healthy reestablishment of HPA-axis reactivity to awakening. This interpretation was supported by inspection of visual plots suggesting that the increase in AUC_I_ for CAR-blunted OC participants was due to a lowered cortisol awakening level (sample 1 taken immediately upon awakening), followed by a more dynamic HPA-axis response to awakening (Additional file 5: Panel S2, c-d).

### Visual attention

Intervention format did not affect changes in the perceptual threshold, *t*_0_, *p* > .6 (Additional file 2: Panel S1, f). The total intervention group improved significantly more than controls on *t*_0_ (*p* < .05, *η*_*p*_^*2*^ = .056) due to significant improvement in OC and no change in controls (Additional file 4: Table S2). A *post hoc* ANCOVA controlling for *t*_*0*_ at baseline, as well as for the other baseline covariates, still supported a significant Time × Group interaction, *F*(1,64) = 5.06, *p* = .028, *η*_*p*_^*2*^ = .073. Additionally, higher OC attendance rates were indicative of larger *t*_0_ improvement, ρ = -.33, *p* = .023. The exploratory analyses of visual short-term memory capacity, *K*, and processing speed, *C*, showed no significant treatment effects, *p*s > .2 (uncorrected). Groups did not differ at any time point or from pre-post treatment in their self-reported motivation to perform the test, *p*s > .1.

## Discussion

Experiences of prolonged psychosocial stress is currently not targeted by evidence-based programs in most public health sectors [45]. The present RCT supported that the meditation-based program “Open and Calm” (OC) developed for this purpose decreased participants’ perceived stress, depressive symptoms, and sleep disturbances, and increased their self-reported mental health and quality of life significantly more than the Danish health sector’s treatment as usual (TAU) for otherwise healthy adults complaining about reduced daily functioning due to prolonged stress. Treatment effects were consistently sustained at 3 months follow-up and OC participants reported significantly better mental health than TAU controls at follow-up on all self-report scales (Table 1). According to well-established cut-offs, OC reduced the risk for depression due to poor quality of life (QOL; [31]) and sleep disturbances (PSQI; [15]). OC participants reported follow-up levels corresponding to Danish norms for perceived stress [71], mental health [5], and symptoms of depression [63] (Fig. 2). Control participants showed heightened risk for depression and suboptimal mental health scores throughout the six months study period.

OC improved the included self-report parameters with similar or slightly larger effect sizes (mean self-report T_1_-T_3_*d* =1.10; mean self-report T_1_-T_2_*d* = 0.70; Table 1) than typically found in studies of healthy samples participating in courses based on mindfulness meditation or transcendental meditation, according to meta-analytic reviews ([21]: *d* = 0.74; [34]: *d* = 0.50; [68]: *d*s = 0.54–0.56). Similarly, the effect from baseline to 3-month follow-up of OC on Cohen’s perceived stress scale (PSS; *d* = 1.30) was larger than a baseline-3-months follow-up analysis on PSS of public stress reduction workshops based on cognitive and/or behavioral therapy ([53], mean *d* = 0.91). Thus, OC seems promising compared with other stress reduction programs. However, the present findings, especially the physiological results, should be interpreted with caution due to the limited sample size compared to meta-analytic reviews. Further and larger OC studies are needed.

Physiological stress, in terms of cortisol secretion and HPA-axis dynamics, was also investigated. The primary analyses included all participants and did not show significant changes on any cortisol outcomes in any group (Additional file 4: Table S2). However, based on the potential exhaustion of HPA-axis dynamics after prolonged stress ([6]) and associations between burnout and blunted CAR [43, 54, 59, 67], our secondary analyses separated participants into two subgroups according to their baseline CAR (AUC_I_) profile. The first subgroup included all participants with an initially present (non-blunted) CAR. Within this group, OC participants decreased significantly on the magnitude of cortisol secretion (AUC_G_), and also significantly more than in non-blunted TAU controls, even after controlling for relevant covariates and baseline AUC_G_ levels. Decreasing circulating levels of cortisol may be important in restoring health and preventing negative consequences of prolonged stress, e.g., because it may prevent neural atrophy in frontal and hippocampal regions, improving top-down regulation of limbic systems, promoting stress resiliency [19]. The stimulated HPA-axis output (AUC_I_) also decreased significantly in OC participants with non-blunted baseline CAR (Additional file 5: Panel S2, a). This change may relate to improved stress resiliency, since HPA-axis reactivity has been associated with several risk factors for depression, including 5-HTTLPR genotype [20]. In the present study, 5-HTTLPR genotype was unrelated to any treatment effects. This is contrary to one study indicating stronger physiological stress reduction effects in SS-carriers than SL-carriers in a student sample [61]. More knowledge is needed on genetic effect moderators of stress reduction effects of meditation-based programs in different sample types. Effects of 5-HTTLPR may decrease with age ([75]). In addition, CAR is not a direct measure of individuals’ reactivity to everyday stressors (but see [20, 32]) and effects of 5-HTTLPR-genotype on reactions to stressful stimuli interact with environmental factors [16]. AUC_I_ changes did not differ between OC and TAU. Thus, the main cortisol effect of OC was a reduction in the magnitude of cortisol secretion for participants with a non-blunted CAR at baseline. In addition, as we hypothesized, AUC_I_ increased significantly for OC participants with a blunted baseline CAR. This suggests that HPA-axis dynamics, i.e., HPA axis reactivity to stimulation (awakening), was re-established (Additional file 5: Panel S2, b). A renormalization of CAR potentially also influenced TAU controls, but we could not evaluate this AUC_I_ change statistically with only *n* = 2 blunted TAU controls (Additional file 4: Table S2). Cortisol studies of meditation and stress reduction have produced mixed findings and lacked methodological rigor [55], rendering the present analytic strategy potentially applicable to future studies of prolonged stress. HPA-axis reactivity (AUC_I_) has been suggested as a potential marker of physiological reactivity to stressors, such as psychosocial stress [32]. However, cortisol is complexly related with prolonged stress and further studies of HPA-axis dynamics, prolonged stress, and burnout are clearly needed [25].

### Visual perception

The threshold of conscious visual perception, *t*_0_, improved significantly more in OC than in controls, also when adjusting for baseline. Further, larger *t*_0_-improvements were associated with increased OC compliance. This corroborates the previously reported finding that the TVA *t*_0_ parameter was specifically improved by meditation and not by physical stress reduction [42]. Interestingly, the TVA-model [12, 13] states that *t*_0_ improvements may reflect stronger reliance on bottom-up-driven perception, rather than conscious recalibration of attentional weights. OC may therefore have improved the perceptual threshold because participants became less prone to consciously modulate visual attention. This aligns with the OC training in *relaxed* and *receptive* (“Open”) awareness of sensory information and a *non-intervening* (“Calm”) conscious witnessing. As mentioned, these are essential elements for many meditative traditions. Correspondingly, the visual perceptual threshold was also improved by yoga [8, 78] and mindfulness meditation [42, 52]. Mindfulness has also improved the threshold for conscious registration of proprioceptive stimuli [62] and the perceptual threshold in an auditory temporal discrimination task [27]. As argued by recent theories, meditation may facilitate insight into personal states and promote objective perception in general through increased perceptual sensitivity within several sensory modalities, i.e., through a lowering of the stimulation needed for conscious registration [4, 14]. Our findings support these proposals, but clearly more research on bottom-up perceptual effects of meditation is needed.

### Experiences from the practical implementation

The Open and Calm program received a 94 % completion rate. GPs found it easy to use a simple, online referral system and maintaining the full screening at the program distributor (Copenhagen University Hospital) ensured similar inclusion procedures throughout. However, among 20 referring GPs, ten GPs referred only one—two patients each. GPs and psychiatrists are generally not accustomed to referring stressed, but otherwise healthy individuals to treatment [57]. To achieve sustainability, we reiterate recommendations [45] that mental health program distributors employ health workers specifically for sustaining recruitment through local health facilities.

The two intervention formats (individual/groups of *n* = 8) yielded similar treatment effects (Additional file 2: Panel S1). This is important, since individual courses required 2.6 times more professional contact hours per participant. Workshops for even larger groups also reduced stress [10, 53] and anxiety [9]. A stepped care model [26] may be recommendable, where less intensive or demanding group programs are offered as a first-line treatment, while smaller or individual courses are offered when deemed necessary. A less intensive (minimal contact) group OC intervention is currently investigated. In general, more systematic research is needed on public health intervention formats [45]. The OC intervention differs from other programs mainly because it was specifically designed for public mental health promotion in a broad demographic group (A full intervention description can be supplied by request to the first author). OC was thus carried out in a health promotion clinic, not in e.g., hospital settings. OC prioritizes everyday words such as *Open* and *Calm*, rather than e.g., *beginner’s mind*, *non-judgment*, or *mindfulness* [44]. Perhaps most importantly, OC teaches meditation as a definable strategy and not as a special state of mind (for a discussion of these contrasting approaches, see Shapiro & Walsh [69]). OC finally emphasizes a body-psycho-social focus on promoting mental health, rather than a more narrow focus on meditation. Therefore, findings may not be generalizable to other types of meditation-based programs.

The dropout rate of only 6 % may be important. Dropout in meditation-based stress reduction programs typically ranges 15-30 % [64]. Based on participant feedback, the most appreciated elements of the OC program were the meditative practices and the programme structure, repeating bodily, mental, and social themes. This, however, is speculative and should be clarified by qualitative studies. We speculate that the choice of conducting evening sessions also lowered the dropout rate, especially for employed OC group participants, enabling them to maintain a normal working week. Individual OC participants could flexibly book course sessions in expanded working hours (8 am–6 pm).

Limitations of the RCT include the need for studying longer time periods, such as a year. A longer study period would enable more direct health impact assessments [49], such as measures of the occurrence of stress-related depression or days of stress-induced absence from work. An active control group would have improved the ability to detect OC-specific effects. However, an unrestricted TAU design allowed a comparison of OC with the current, unsystematic treatments offered for healthy adults dealing with prolonged stress. As another limitation, the paucity of significant associations between OC session attendance rates and outcome change scores should not be extrapolated to indicate that compliance with meditation is unimportant in OC or similar programs. The absence of evidence is not the evidence of absence – and several studies of short-term meditation programs did find that increased meditation compliance was related to larger treatment effects [79]. The simple compliance measure of session attendance may have prevented us from detecting such associations. More detailed compliance measures are most likely necessary to illuminate the importance of compliance with different elements of such programs. Relatedly, our relatively low sample size, especially in the cortisol analyses, limits the statistical power to detect treatment effect moderators, so these secondary findings should also be interpreted cautiously.

## Conclusion

This RCT revealed that the OC program designed specifically for public stress reduction and mental health promotion improved self-reported stress, depressive symptoms, sleep disturbances, mental health, and quality of life, a physiological stress marker (the magnitude of cortisol secretion), and the threshold for visual perception significantly more than treatment as usual for Danish, stressed adults. The program participant completion rate was 94 %. All self-report effects were sustained or further improved at 3 months follow-up. We found no consistent effect moderation by age, gender, education, 5-HTTLPR-genotype, or any other control variables, while higher trait harm-avoidance might attenuate effects of OC. In sum, the OC program was consistently supported as effective. Further testing of potential advantages, including long-term more direct health sectorial benefits, of the OC program is therefore warranted. Due to the positive results, a larger implementation of the OC program is in progress in the health promotion sector in the municipality of Copenhagen.

## Additional files

Additional file 1: Table S1. Sample characteristics. (DOCX 24 kb) Additional file 2: Panel S1. Comparisons of interventional formats on self-report and visual perception. (PDF 72 kb) Additional file 3:
**Supplementary findings: Potential effect moderation by TCI-Harm Avoidance.** (DOCX 119 kb) Additional file 4: Table S2. Treatment effects on cortisol and visual attention. (DOCX 29 kb) Additional file 5: Panel S2. Open and Calm participants’ changes in cortisol secretion dynamics. (PDF 60 kb)
