# Supplementary material for: Open and Calm – A randomized controlled trial evaluating a public stress reduction program in Denmark
Source: BMC Public Health. 2015 Dec 16;15:1245. doi: 10.1186/s12889-015-2588-2 (PMC4682248; doi:10.1186/s12889-015-2588-2)
Supplement: Additional file 1: Table S1. — Sample characteristics. (DOCX 24 kb) [file 12889_2015_2588_MOESM1_ESM.docx]

| **Supplementary table 1**. Sample characteristics | | | | | | | | | | | | |
| --- | --- | --- | --- | --- | --- | --- | --- | --- | --- | --- | --- | --- |
| Measures | | | TAU | | | OC-I | | | OC-G | | Comparison | |
| *Demographics and health variables* | | *%* | | *(n)* | *%* | | *(n)* | *%* | | *(n)* | | *p* |
|  | Gender (women) | 58.33 | | (14) | 70.77 | | (17) | 66.67 | | (16) | >.6 | |
|  | Employment (employed) | 91.70 | | (22) | 79.20 | | (19) | 91.70 | | (22) | >.3 | |
|  | Smokers (% daily smokers) | 12.50 | | (3) | 0 | | (0) | 4.17 | | (1) | >.1 | |
|  |  |  | |  |  | |  |  | |  |  | |
|  |  | *%* | | *(n)* | *%* | | *(n)* | *%* | | *(n)* |  | |
| Meditation Experience (% yes^a^) | | 8.33 | | (2) | 12.50 | | (3) | 4.17 | | (1) | >.5 | |
|  | |  | |  |  | |  |  | |  |  | |
|  | | *Mean* | | *(SD)* | *Mean* | | *(SD)* | *Mean* | | *(SD)* |  | |
|  | Age (years) | 42.58 | | (7.19) | 42.46 | | (9.21) | 41.67 | | (10.38) | >.9 | |
|  | Professional education | 3.71 | | (1.27) | 3.21 | | (1.38) | 3.42 | | (1.44) | >.4 | |
|  | Body-Mass-Index | 24.96 | | (2.82) | 25.53 | | (3.20) | 23.88 | | (2.72) | >.1 | |
|  | Alcohol consumption (units/week) | 4.87 | | (4.11) | 3.02 | | (2.01) | 4.21 | | (3.46) | >.5 | |
|  |  |  | |  |  | |  |  | |  |  | |
| *5-HTTLPR genotype* | | *%* | | *(n)* | *%* | | *(n)* | *%* | | *(n)* | | *p* |
|  | 0 LA-alleles (SS/SLG) | 16.67 | | (4) | 25.00 | | (6) | 25.00 | | (4) |  | |
|  | 1 LA-allele (SLA/LGLA) | 54.17 | | (13) | 45.83 | | (11) | 70.83 | | (17) | >.3 | |
|  | 2 LA-alleles (LALA) | 25.00 | | (6) | 29.17 | | (7) | 8.33 | | (2) |  |  |
|  | Missing | 4.17 | | (1) | 0.00 | | (0) | 4.17 | | (1) |  | |
|  | |  | |  |  | |  |  | |  | |  |
| *Psychological background variables* | | *Mean* | | *(SD)* | *Mean* | | *(SD)* | *Mean* | | *(SD)* | | *p* |
|  | Stressful life events (past year) | 4.21 | | (2.95) | *4.96* | | *(2.89)* | *4.21* | | *(3.58)* | >.6 | |
|  | Stressful life events (lifetime) | 2.29 | | (1.52) | *2.75* | | *(1.7)* | *2.54* | | *(1.44)* | >.4 | |
|  | TCI Self-Directedness (TCI-SD) | 26.33 | | (8.80) | 29.38 | | (7.48) | 30.92 | | (7.91) | >.1 | |
|  | TCI Harm Avoidance (TCI-HA) | 19.63 | | (11.25) | 20.25 | | (9.99) | 23.04 | | (11.8) | >.4 | |
|  | Attentional instability (MAAS) | 3.77 | | (0.55) | 3.62 | | (0.77) | 3.90 | | 0.68 | >.1 | |
| *Notes*. *p*-values are two-tailed, uncorrected for multiple tests. OC-I = Open and Calm – Individual format. OC-G = Open and Calm – Group format. TAU = Treatment As Usual. Professional education is scored from 1—5: 1= no professional education, 2 = 1-2 years, 3 = 2-3 years, 4 = 3-4years, 5 = >4 years. TCI = Temperament and Character Inventory. MAAS = Mindful Attention Awareness Scale. ^a^. Meditation experience was defined as having meditated > 2 times per week for > one month. | | | | | | | | | | | | |
