# Supplementary material for: Open and Calm – A randomized controlled trial evaluating a public stress reduction program in Denmark
Source: BMC Public Health. 2015 Dec 16;15:1245. doi: 10.1186/s12889-015-2588-2 (PMC4682248; doi:10.1186/s12889-015-2588-2)
Supplement: Additional file 2: Panel S1. — Comparisons of interventional formats on self-report and visual perception. (PDF 72 kb) [file 12889_2015_2588_MOESM2_ESM.pdf]

Supplementary panel 1. Comparisons of interventional formats on self-report and visual perception

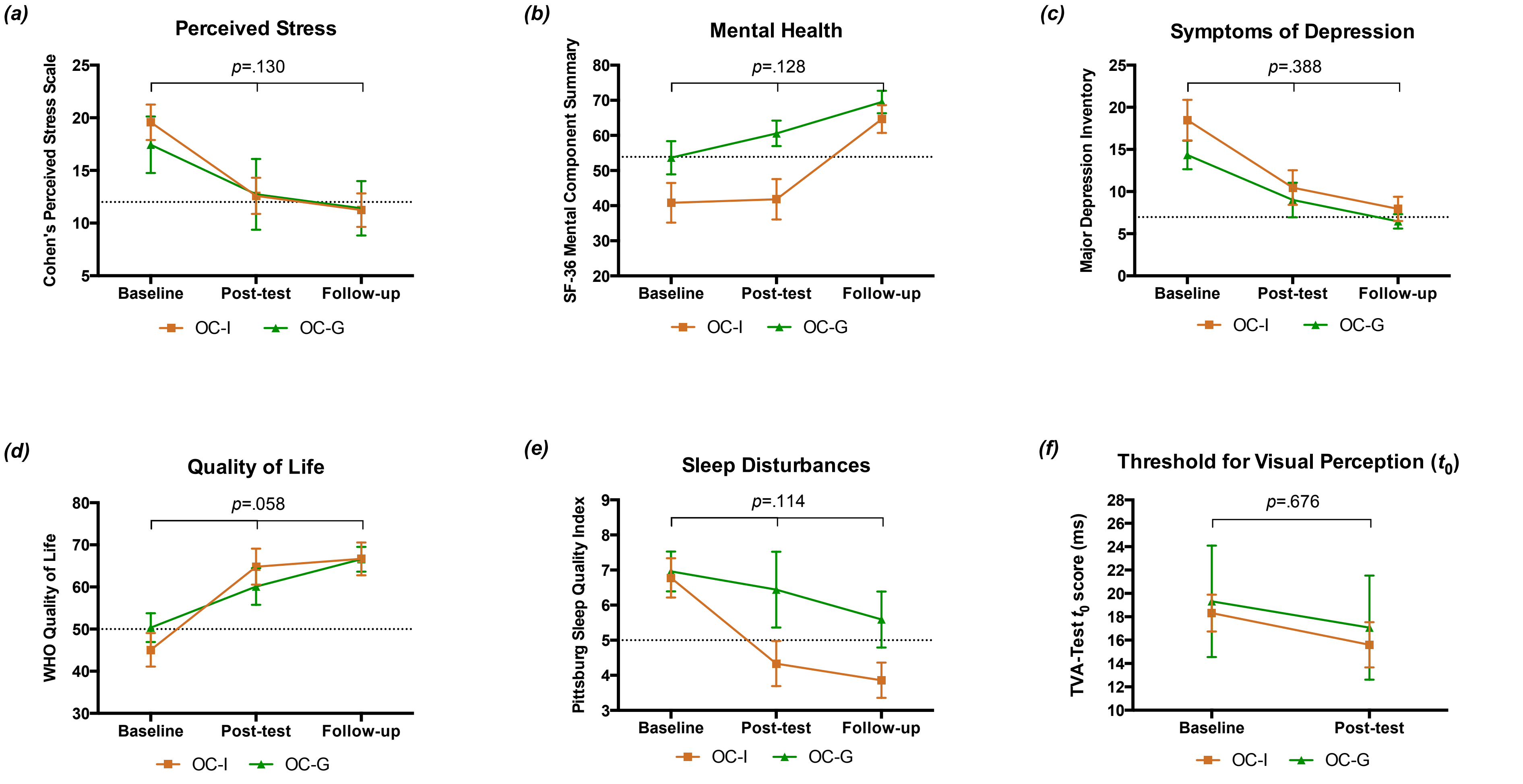

Notes. *.*p*<.05.**.*p*<.01.***.*p*<.001. *p*-values are two-tailed, corrected for multiple tests (Bonferroni-Holm), and based on intent-to-treat-analyses (Open and Calm [OC] *N*=48. Treatment As Usual [TAU] *N*=24) after adjustment for relevant biological, socioeconomic, and psychological trait variables. Asterisks (*) above horizontal lines represent *p*-values of Time*Group effects, while asterisks or *p*-values above error bars represent p-values of between-group comparisons (Table 2). Error bars represent 95% CI of the mean. ***(a).*** The dotted line represent the mean among a national region-stratified random sample of >21.000 Danish adults (Stigsdotter et al., 2010). ***(b)*** The dotted line represents the age-adjusted Danish norm for the SF36-Mental Health Component (Bjørner et al., 1997) ***(c).*** The dotted line represents the Danish norm (Olsen et al., 2004). ***(d)*** Scores below the dotted line represent a risk marker for depression (Folker & Folker, 2008). ***(e)*** Scores above the dotted line represent a risk marker for depression (Buysse et al., 1989). As seen, OC-I shows descriptively (but not significantly) larger improvement on sleep disturbances than OC-G. ***(f)*** Changes in the threshold for visual perception, t_0.
