# Supplementary material for: Open and Calm – A randomized controlled trial evaluating a public stress reduction program in Denmark
Source: BMC Public Health. 2015 Dec 16;15:1245. doi: 10.1186/s12889-015-2588-2 (PMC4682248; doi:10.1186/s12889-015-2588-2)
Supplement: Additional file 3: — Supplementary findings: Potential effect moderation by TCI-Harm Avoidance. (DOCX 119 kb) [file 12889_2015_2588_MOESM3_ESM.docx]

**­Open and Calm – A randomized controlled trial evaluating a public stress reduction program in Denmark**

## *Supplementary findings: Potential effect moderation by TCI-Harm Avoidance.*

Corresponding author

Christian Gaden Jensen: [cgj@cfps.dk](mailto:cgj@cfps.dk)

## Effect Moderation results

Table 1 and Supplementary table 2 of the full manuscript display all included covariates. We here only report on the covariates that were supported as significant moderators of outcome changes, as indicated by significant (*p*<.05, uncorrected for multiple tests) Time × Group × covariate interactions. The most consistent moderator of outcome changes was TCI Harm Avoidance. For the magnitude of cortisol secretion, AUC_G_, the repeated measures ANCOVA across all (*n*=47) participants revealed a significant Time × Group × TCI-HA interaction, *F*(2,44)=3.85, *p*=0.029, *η_p_^2^*=.15. This was also significant in the subgroup AUC_G_-analysis of non-blunted participants, *F*(2,22)=5.83, *p*=.009, *η_p_^2^*=.35. In both the full sample and the non-blunted sample, the correlation screenings revealed that higher TCI Harm Avoidance scores were associated with smaller decreases in cortisol (AUC_G_) in TAU controls, *ρ*s=.58-.59, *p*s ≤ .035. TCI-HA scores were unrelated to AUC_G_ and AUC_I_ changes within both the full, the CAR-non-blunted, and the CAR-blunted OC samples, *p*s>.26. These results, as well as inspection of visual plots of AUC_G_-changes when splitting TAU and OC groups at the TCI-HA median, suggested that a more avoidant personality was a stronger disadvantage for TAU controls than for OC participants with respect to decreasing cortisol (the Time × Group × TCI-HA interaction), since higher TCI avoidance scores were indicative of smaller AUC_G_ decreases in TAU only. For CAR-non-blunted participants, we also found a Time × Group × Age interaction in the AUC_G_-analyses, *F*(2,22)=8.57, *p*=.002, = .44. Correlation tests revealed that higher age was associated with larger AUC_G_ increases in OC (*r*=.58, *p*=.025), while this was not found in TAU controls, but there was a tendency in the same direction, *r*=.51, *p*=.078. These findings, as well exploratory visual plots of AUC_G_ changes in OC and TAU groups split at the age median, suggested that higher age might have represented a slightly larger advantage with respect to reducing total cortisol secretion for OC participants than for the TAU controls.

Effect moderation for self-report changes further supported a role for TCI-HA. For quality of life changes, the Time × Group × TCI-HA interaction was significant, *F*(4,132)=2.54, *p*=.043, *η_p_^2^*= .07. Correlation tests revealed that high harm avoidance was related to less long-term improvement on QOL in OC participants (*ρ*=.29, *p*=.054), but not in controls, *r*=.08, *p*>.7. Visual plots supported that OC participants with high harm avoidance at baseline improved less on QOL than OC participants with low (median split) TCI-HA, while this difference was not found in TAU.

Thus, again, an initially more avoidant personality seemed to decrease effects of OC. Two other three-way interactions were found. First, higher BMI seemed to be a stronger factor for improvement on QOL in controls (*r*=.50, *p*=.023) than in OC participants (*ρ*=-.16, *p*=30), rendering the Time × Group × BMI interaction significant, *F*(4,132)=2.54, *p*=.041, *η_p_^2^*= .07. Second, daily smokers improved more on PSQI than non-smokers within TAU (*ρ*=-.51, *p*=.015), but not in OC (*ρ*=.04, *p*>.7), yielding a Time × Group × smoking interaction, *F*(4,136)=2.82, *p*=.027, *η_p_^2^*= .08. These findings suggested that BMI and smoking played a lesser role for long-term changes in quality of life for participants in the treatment group than for control participants.

For visual attention, TCI-HA scores again seemed to moderate outcome changes differently in the two groups, as indicated by a significant Time × Group × TCI-HA interaction in the exploratory analyses of visual short-term memory capacity, *K*, *F*(2,64)=12.18, *p*<.001, *η_p_^2^*= .28. Correlations supported that higher initial avoidance was related to smaller improvements on visual short-term memory in OC participants (*ρ*=.46, *p*=.001), but not in TAU controls, *ρ*=.06, *p*>.7. These results, as well as inspection of visual plots of *K*-changes after splitting the OC and TAU groups at the TCI-HA median, indicated that initially higher avoidance scores represented a stronger inhibiting factor for attaining improvements of the visual short-term memory capacity for OC participants than for controls. For the primary attentional outcome, the threshold for visual perception, *t*_0_, we found a significant Time × Group × Test motivation interaction, *F*(1,68)= 4.48, *p*=.038, *η_p_^2^*= .06. Increases in test motivation were related to larger *t*_0_-improvements in OC participants (*ρ*=.36, *p*=.012), but not in controls, *ρ*=-.01, *p*>.9. These findings, and visual plots, suggested that increased test motivation was a larger factor for perceptual improvements in OC participants than in the controls.

## Discussion

In summary, higher baseline levels of trait harm avoidance, TCI-HA, attenuated treatment effects on self-reported quality of life and visual short-term memory capacity. Higher trait harm avoidance inhibited decreases in cortisol levels in TAU controls. Effect moderation was not a focus of the present RCT, so the present findings should be interpreted with caution. Nonetheless, the TCI-HA findings were rather consistent, and seem meaningful in relation to the core methodology of OC, focusing on training approach-oriented (rather than avoidant) attentiveness and coping. Thus, participants with an initially stronger tendency to use avoidant or repressive coping may have found it less effective or more difficult to apply the approach-oriented OC strategy. This assumption is also in line with the first review of contraindications for mindfulness-based stress reduction, which recommended that harm avoidance was a relevant assessment criterion prior to inclusion in MBSR

(Dobkin et al., 2012). The disposition to by attentive to the present, as measured by the Mindful Attention Awareness Scale in its Danish translation, was also negatively associated with TCI-HA in a large, Danish community sample, *ρ* = -.36 (95% CI [-.28 – -.43]), *p*<.01 (Jensen et al., *in press*). Thus, effects of OC and of MBSR, may be attenuated by higher initial levels of harm-avoidance.

## References

Dobkin, P. L., Irving, J. a., & Amar, S. (2012). For Whom May Participation in a Mindfulness-Based Stress Reduction Program be Contraindicated? *Mindfulness*, *3*(1), 44–50. doi:10.1007/s12671-011-0079-9

Jensen, C.G., Nicklasen, J., Petersen, A., Vangkilde, S., & Hasselbalch, S. G. (*in press*). General Inattentiveness is a Long-term Reliable Trait Independently Predicative of Psychological Health: Danish Validation Studies of Mindfulness Attention Awareness Scale, *Psychological Assessment.*
