# Supplementary material for: Open and Calm – A randomized controlled trial evaluating a public stress reduction program in Denmark
Source: BMC Public Health. 2015 Dec 16;15:1245. doi: 10.1186/s12889-015-2588-2 (PMC4682248; doi:10.1186/s12889-015-2588-2)
Supplement: Additional file 4: Table S2. — Treatment effects on cortisol and visual attention. (DOCX 29 kb) [file 12889_2015_2588_MOESM4_ESM.docx]

| **Supplementary table 2.**  Treatment effects on cortisol and visual attention | | | | | | | | | | | |  |
| --- | --- | --- | --- | --- | --- | --- | --- | --- | --- | --- | --- | --- |
| Outcome | Open and Calm (OC) | | | Treatment As Usual (TAU) | | | OC *vs.* TAU | | OC *vs.* TAU change^a^ | | |  |
|  | *M* | *(SD)* | *d (within)* | *M* | *(SD)* | *d (within)* | *d (between)* | *p* | *F* | *η_p_^2^ (between)* | *p* | Covariates |
| *Cortisol awakening response* |  |  |  |  |  |  |  |  |  |  |  |  |
| All participants |  |  |  |  |  |  |  |  |  |  |  |  |
| AUC-Ground (T_1_) | 1265.01 | (359.21) |  | 1297.22 | (353.42) |  | 0.09 | .774 | 0.14 | .00 | .709 | TCI-HA |
| AUC-Ground (T_2_)^b^ | 1205.70 | (306.80) | -0.27 | 1177.36 | (472.63) | -0.29 | 0.08 | .834 |  |  |  |  |
| AUC-Increase (T_1_) | 127.89 | (405.94) |  | 225.64 | (368.60) |  | 0.25 | .444 | 0.74 | .02 | .394 |  |
| AUC-Increase (T_2_) | 163.63 | (272.97) | 0.12 | 165.28 | (206.09) | 0.16 | 0.01 | .984 |  |  |  |  |
|  |  |  |  |  |  |  |  |  |  |  |  |  |
| Normal baseline CAR |  |  |  |  |  |  |  |  |  |  |  |  |
| AUC-Ground (T_1_) | 1489.40 | (312.94) |  | 1277.30 | (333.05) |  | 0.68 | .094 | 7.04 | .24* | .030 | Age |
| AUC-Ground (T_2_)^b^ | 1339.65 | (299.73) | -0.59* | 1251.23 | (449.28) | -.12 | 0.25 | .541 |  |  |  | TCI-HA |
| AUC-Increase (T_1_) | 436.62 | (315.46) |  | 329.26 | (261.31) |  | 0.38 | .359 | 2.99 | .13 | .099 | Education, |
| AUC-Increase (T_2_) | 269.10 | (237.67) | -0.76* | 175.64 | (192.25) | -.50 | 0.44 | .287 |  |  |  | Gender |
|  |  |  |  |  |  |  |  |  |  |  |  |  |
| Blunted baseline CAR |  |  |  |  |  |  |  |  |  |  |  |  |
| AUC-Ground (T_1_) | 1078.02 | (284.22) |  | 1426.71 | (615.59) | -^c^ | - ^c^ | - ^c^ | - ^c^ | - ^c^ | - ^c^ | - |
| AUC-Ground (T_2_) ^b^ | 1094.08 | (272.35) | 0.08 | 697.23 | (416.05) | - ^c^ | - ^c^ | - ^c^ |  |  |  |  |
| AUC-Increase (T_1_) | -112.22 | (291.29) |  | -396.09 | (336.46) | - ^c^ | - ^c^ | - ^c^ | - ^c^ | - ^c^ | - ^c^ | - |
| AUC-Increase (T_2_) ^b^ | 81.60 | (276.40) | 0.88* | 103.11 | (369.51) | - ^c^ | - ^c^ | - ^c^ |  |  |  |  |
|  |  |  |  |  |  |  |  |  |  |  |  |  |
| *Visual attention* |  |  |  |  |  |  |  |  |  |  |  |  |
| Perceptual threshold, *t_0_* (T_1_) | 18.82 | (9.14) |  | 16.40 | (6.39) |  | 0.29 | .531 | 4.37 | .06* | .041 | Education |
| Perceptual threshold, *t_0_* (T_2_) ^b^ | 16.34 | (9.75) | -0.35* | 17.12 | (9.81) | 0.15 | 0.08 | .445 |  |  |  | Motivation |
| STM capacity, *K* (T_1_) | 2.67 | (0.57) |  | 2.78 | (0.67) |  | 0.18 | .501 | 0.14 | .00 | .714 |  |
| STM capacity, *K* (T_2_) ^b^ | 2.74 | (0.65) | 0.12 | 2.82 | (0.74) | 0.06 | 0.12 | .651 |  |  |  |  |
| Processing speed, *C* (T_1_) | 50.60 | (17.16) |  | 50.57 | (16.24) |  | 0.00 | .995 | 0.89 | .01 | .348 |  |
| Processing speed, *C* (T_2_) ^b^ | 51.85 | (17.82) | 0.07 | 55.53 | (16.53) | 0.29 | 0.21 | .401 |  |  |  |  |
| *Notes*. *.*p*<.05.**.*p*<.01.***.*p*<.001. All *p*-values are two-tailed and based on intent-to-treat-analyses (all OC *n*=34; all TAU *n*= 15; non-blunted OC *n*=15; TAU *n*=13; blunted OC *n*=18; blunted TAU *n*=2). ^a^. Analyses of OC *vs*. TAU changes were adjusted for relevant covariates as listed in the column ‘Covariates’, *p*-values were Bonferroni-Holm corrected. and effect sizes indicate pre-treatment—post-treatment Time ✕ Group effects.^b^. Within-group effect sizes indicate pre-treatment—post-treatment effects adjusted for dependence among means (Morris & Deshon, 2002, formula 8).^c^.Test not conducted since TAU *n*=2. Motivation = Change scores for self-reported motivation to perform the TVA test (see main text). TCI-HA = Temperament and Character Inventory – Harm Avoidance. | | | | | | | | | | | | |
