# Supplementary material for: Open and Calm – A randomized controlled trial evaluating a public stress reduction program in Denmark
Source: BMC Public Health. 2015 Dec 16;15:1245. doi: 10.1186/s12889-015-2588-2 (PMC4682248; doi:10.1186/s12889-015-2588-2)
Supplement: Additional file 5: Panel S2. — Open and Calm participants’ changes in cortisol secretion dynamics. (PDF 60 kb) [file 12889_2015_2588_MOESM5_ESM.pdf]

Supplementary Panel 2. Open and Calm participants' changes in cortisol secretion dynamics

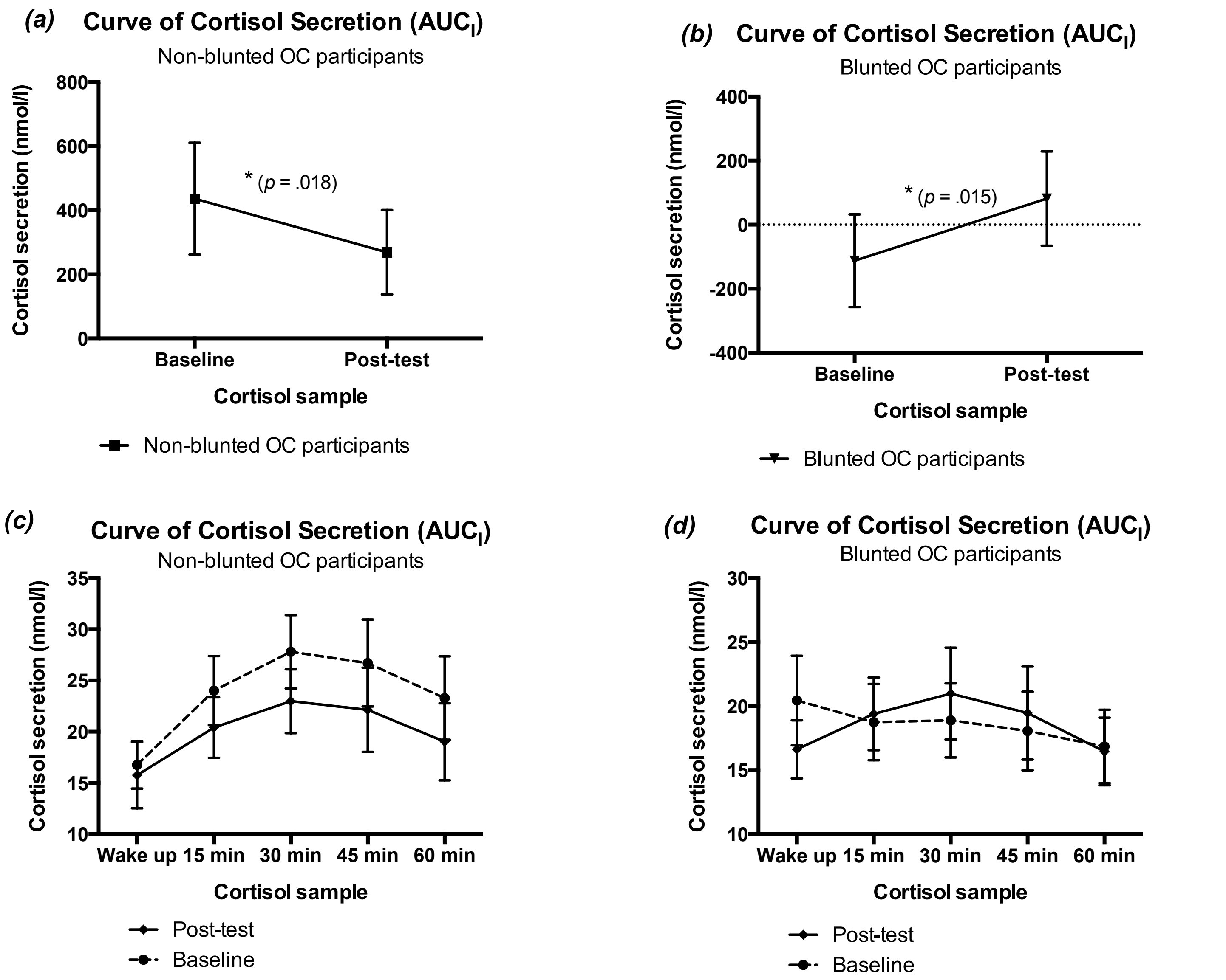

Notes.*.*p*<.05 (two-tailed, intent-to-treat analyses). ***(a)***. Pre-post changes in the Cortisol Awakening Response (CAR) Area Under Curve - Increase (AUC_I) for non-blunted OC participants (*n*=15). ***(b)***. Pre-post changes in AUC_I for blunted OC participants (*n*=18). ***(c)***. Descriptive plot of baseline and post-test values for saliva samples 1-5 for non-blunted OC participants. ***(d)***. Descriptive plot of baseline and post-test values for saliva samples 1-5 for blunted OC participants. Error bars represent 95% CI of the mean. Supplementary Table 2 shows descriptive cortisol data and test statistics.
